# Concussions analysis in 2022–2024 CONMEBOL soccer tournaments

**DOI:** 10.3389/fneur.2025.1645543

**Published:** 2025-09-30

**Authors:** Osvaldo Pangrazio, Francisco Forriol, Alex S. Aguirre, Tina Bastin, Alcy R. Torres

**Affiliations:** ^1^Department of Orthopedics, South American Football Confederation (CONMEBOL), Luque, Paraguay; ^2^Department of Family Medicine, South American Football Confederation (CONMEBOL), Luque, Paraguay; ^3^Department of Pediatrics, Boston Medical Center, Boston University Chobanian and Avedisian School of Medicine, Boston, MA, United States

**Keywords:** concussion, trauma, brain, soccer, protocol, global

## Abstract

**Introduction:**

Sports-related concussions (SRC) are a pressing global health concern. However, countries in the Global South often lack standardized diagnostic criteria, and limited medical resources lead to inconsistent SRC detection. Our study presents a culturally adapted concussion detection protocol implemented by the region’s governing soccer federation, CONMEBOL.

**Methods:**

The Concussion Fast Recognition Protocol (CFRP) was developed by adapting the Sport Concussion Assessment Tool, the Standardized Assessment of Concussion, and incorporating community-level input to ensure cultural relevance. The protocol was written in English, Spanish, and Portuguese. On-field medical teams completed the CFRP after each CONMEBOL match (*N* = 156) from 2022 to 2024, regardless of whether an SRC occurred. Players included in the study (*N* = 5,928) ranged in age from 15–44 years.

**Results:**

A total of 27 concussions were identified, all of which were immediately removed from play. Most concussions (37.04%) occurred during the 21–45-min period, and 59.26% involved away-team athletes. The incidence rate per 1,000 player-hours was 2.61. Game temperatures ranged from 7 °C to 36 °C, and the maximum altitude reached 4,150 meters. The distance covered by players ranged from 550 to 6,100 meters. Incidence rates per 1,000 player-hours were calculated.

**Discussion:**

This first large-scale, prospective SRC study in South American soccer demonstrates effective implementation of a regional protocol and identifies context-related trends in SRC risk. The findings underscore the importance of a culturally appropriate protocol and contribute novel data to global concussion literature.

## 1 Introduction

**Figure 1:**
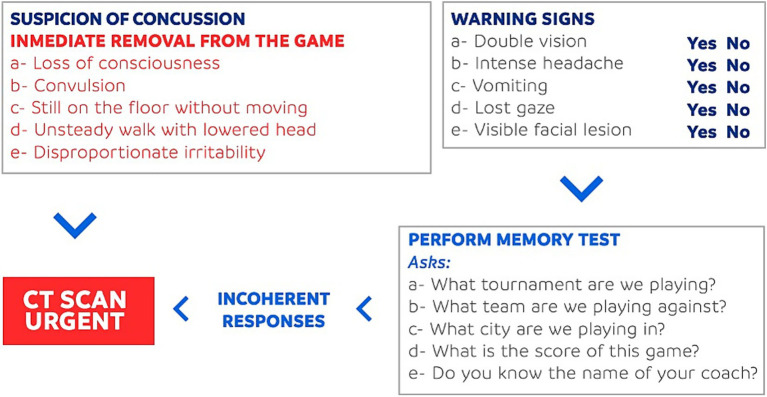
CFRP protocol by CONMEBOL.

Sports-related concussions represent a growing public health concern, particularly in soccer which is a sport with global participation and popularity (1, 2). Concussions account for approximately 22% of all soccer-related injuries, highlighting the substantial risk of head trauma despite the physical and mental health benefits of athletic engagement (1, 3). Concussion is defined as a traumatic brain injury that results in transient alterations in brain function, often caused by biomechanical forces. In soccer, concussions most commonly result from player-to-player contact, but may also occur due to collisions with the playing surface or equipment (1, 3). Concussions fit into the milder form of classification of a traumatic brain injury (TBI) with usually a Glasgow Coma Scale (GCS) score of 13–15. Lower scores of GCS are considered moderate or severe TBI and do not fit into a concussion definition (4). Figure 1 shows the red flags and warning signs on the CFRP protocol created by the Medical Commission of CONMEBOL to guide management of concussions (5).

Despite growing awareness, the true incidence of concussion remains underestimated (6). Current surveillance systems, such as those by the Centers for Disease Control and Prevention (CDC), primarily track cases involving loss of consciousness, a symptom present in fewer than 10% of all concussions, leading to under recognition and underreporting (7). The short and long-term consequences of concussion are well documented, ranging from impairments in cognitive processing and emotional regulation to delayed physical recovery (8). Notably, athletes who continue to play after sustaining a concussion face significantly longer recovery times, with delays in removal from play linked to prolonged symptom duration and increased risk of further injury (9, 10). These findings underscore the critical need for timely recognition and appropriate management of concussions in sport.

To address this, standardized concussion protocols have been developed and implemented at both national and international levels. Tools such as the Sport Concussion Assessment Tool (SCAT6) provide structured guidelines for identification, removal from play, and return-to-play decisions (11). In South America, the Confederación Sudamericana de Fútbol (CONMEBOL) introduced the Concussion Fast Recognition Protocol (CFRP) to improve on-field concussion detection and management in professional soccer (8, 12). Despite these advancements, the successful implementation of concussion protocols remains inconsistent across regions, often hindered by limited medical resources, lack of culturally and linguistically validated tools, and variable awareness among stakeholders. This study aims to evaluate the application and efficacy of the CFRP in South American soccer and correlate it with data from international leagues to generate informed decisions in the future.

## 2 Methods

Concussions from CONMEBOL Libertadores and Sudamericana tournaments were registered prospectively from the beginning to end of those tournaments during 2022 to 2024. A total of 156 games were analyzed retrospectively from the data collected to understand the concussion rates in professional soccer tournaments and consequently identify players adherence to concussion protocols (Table 1). The concussions were identified in each game by the referee, and the information was shared with the medical board of CONMEBOL using the Concussion Fast Recognition Protocol, a tool to report concussions validated by this organization. Additionally, each team’s physician staff signed a document confirming the concussion and agreeing on following the concussion protocol. A CFRP report was completed after every match even if no concussion was identified.

**Table 1:** Characteristics of study population.

Characteristics	Data	
Sample	*n* = 5,928
Gender	Male (100%)	Female (0%)
Age distribution (years)	15–44	
Age mean (years)	31	
Level of play	First division (100%)	

If a concussion was suspected, the team’s health staff evaluated the player. The injured player was removed from the game when necessary, and a report describing all the incident details was filed. The data were analyzed in relationship with international data to provide a comprehensive review of how soccer protocols are typically applied.

## 3 Results

**Figure 2:**
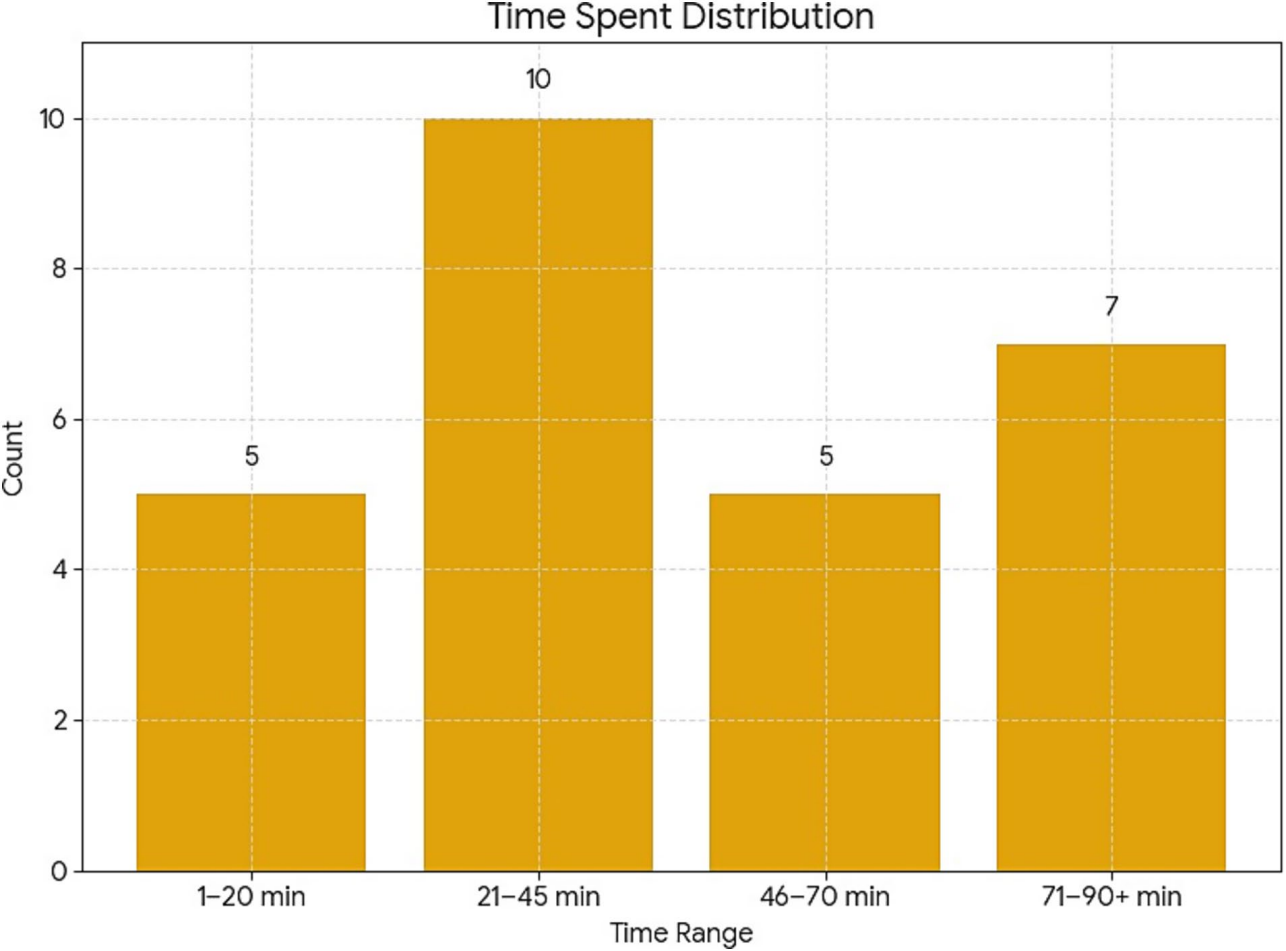
Concussion numbers according to period of the game.

**Figure 3:**
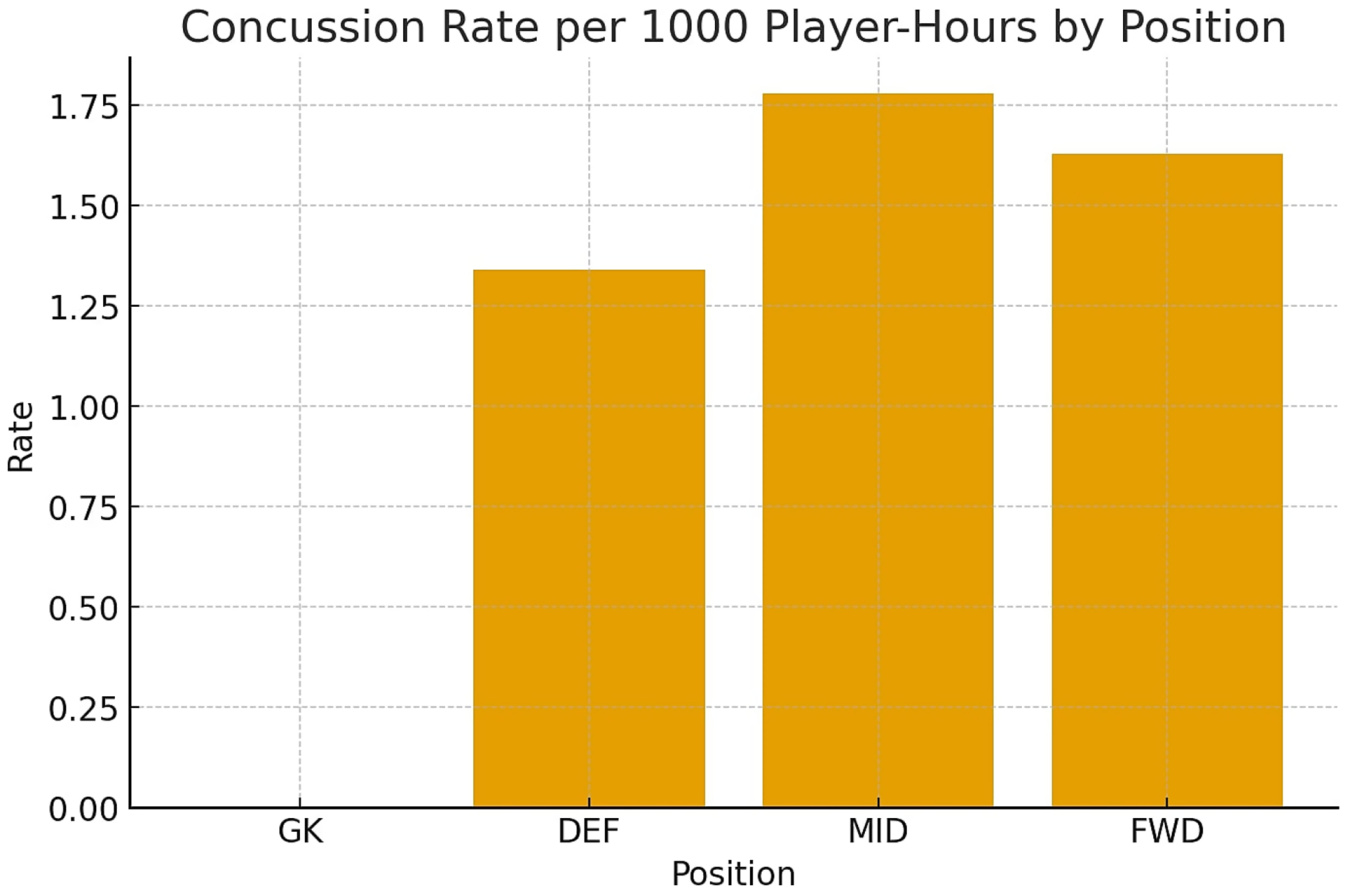
Concussion rates per 1,000 player-hours by specific position (GK, goalkeeper; DEF, defenses; MID, midfielders; FWD, forwards).

A total of 27 concussions were identified. All concussed players were removed from the field following the protocol. There were 145 teams that played over 930 games with 5,928 players. Most of the games were played between March and May, where most concussions happened compared to later stages of the tournaments (October to November). The temperature in the tournament ranged from 7 °C to 36 °C. The highest altitude for a game was 4,150 m in El Alto, Bolivia. The distance that players had to run ranged from 550 to 6,100 meters. The distance was measured by multiple camera systems with specialized software. The age of the players ranged from 15 to 44. In general, there were a higher number of concussions in the first half of games (16 compared to 11 in the later half), specifically close to the half time between minutes 21–45 as seen in Figure 2. There were 12 concussions in midfielders, 9 concussions in defenses, and 5 concussions in forwards as seen in Table 2 and Figure 3. The data show a higher number of concussions in the visitor side (16 compared to 11 in the home team). The concussion rate per 1,000 player-hours was 2.61. There was no specific team or city where concussions happened more frequently.

**Table 2:** Concussion rates per 1,000 player-hours by position (GK: goalkeeper, DEF: defenses, MID: midfielders, FWD: forwards).

Position	Total players	Assigned concussions	Rate (per 1,000 player-hours)
GK	539	0	0
DEF	2,151	9	1.34
MID	2,151	12	1.78
FWD	1,087	5	1.63
Total	5,928	26	2.61

## 4 Discussion

This study underscores the critical and growing public health concern of sports-related concussions in professional soccer, particularly within South American tournaments governed by CONMEBOL. The implementation of the Concussion Fast Recognition Protocol (CFRP) appears to have contributed positively to the identification and management of concussion events. Out of all the games, only 156 games were analyzed because these were the only games included the CFRP. From the 156 games, 27 concussions were identified, yielding a concussion rate of 2.61 per 1,000 player-hours which is comparable to the upper ranges reported in leagues such as Major League Soccer (13.48 per 1,000 player-hours was calculated from article data, 20.22 per 1,000 athlete-exposures reported) and the English Premier League (12.45 per 1,000 player-hours was calculated from article data, 18.68 per 1,000 athlete-exposures reported), and significantly higher than figures from French men’s football (0.44 per 1,000 player-hours were reported) and Swedish elite soccer (1.19 per 1,000 player-hours were reported) (13–16).

Encouragingly, all concussions observed in this study were managed in accordance with CFRP guidelines, including timely removal from play and appropriate documentation. Not following protocols or allowing players to continue playing has been evidenced in other leagues such as the professional French football where a review revealed a consistent trend of disregard for concussion management guidelines. In the review of the French league, more than half (53%) of concussed players went back to the game, and over a quarter (28%) were not even medically evaluated on the field. This behavior goes against official recommendations from governing bodies (14). Nevertheless, this study reflects a growing adherence to the protocol and stands in contrast to findings from other leagues (13–16). Similarly, during the 2016 UEFA European Championship, only 27.5% of potential concussive events (PCEs) were medically evaluated, despite most of those players exhibiting clear concussion symptoms (13).

These findings emphasize that the existence of standardized protocols alone is insufficient; effective implementation, accountability, and a culture of safety are equally essential. The American Medical Society for Sports Medicine (AMSSM) has advocated for strict adherence to evidence-based concussion protocols, including rapid sideline assessment and a supervised, graduated return-to-play process (17). However, studies consistently show gaps in compliance across multiple elite leagues, suggesting that awareness and infrastructure may be lagging behind clinical guidance (13–15).

The current study also revealed patterns that may inform injury prevention strategies. A greater number of concussions occurred in the first half of games, especially between minutes 21–45, which may relate to the physical escalation of gameplay or a transitional period where mental and physical fatigue begin to emerge. There was also a higher number of concussions among visiting teams, possibly due to travel-related stressors, unfamiliar environments, or differences in acclimatization to temperature and altitude. Notably, the tournament featured different altitude conditions (up to 4,150 meters in El Alto, Bolivia) and temperatures ranging from 7 °C to 36 °C, yet no specific environmental variable appeared directly correlated with increased concussion risk, though further research with larger datasets may clarify these relationships.

The distribution of injuries by position also aligns with global data, where defenders and midfielders who are frequently involved in high-contact and high-intensity scenarios tend to have higher injury rates (18–20). Furthermore, the occurrence of increased number of concussions near the end of matches compared to the beginning of the second half suggests that fatigue and decreased vigilance may elevate risk, pointing to a need for additional medical monitoring during the closing stages of play (18, 19). This could include increasing the availability of medical staff during late-game periods or enforcing mid-game screening tools for players involved in high-impact events.

Limitations of this study include reliance on referee and medical staff identification for initial concussion recognition, which may allow some cases to go undetected, especially in the absence of loss of consciousness, which occurs in less than 10% of concussions (7). Additionally, while protocol adherence was documented, the post-removal care quality and return-to-play compliance were not assessed, which are essential components of effective concussion management. Another limitation was the retrospective analysis that does not include a follow up of the players with concussions. The sample size and gender (only male players) also limit generalizability, and the observational design precludes causal inference.

Future research should explore longitudinal follow-up of concussed athletes to assess long-term outcomes, protocol compliance over time, and the impact of educational interventions for coaching staff and referees. Integrating technology such as sideline video replay and head impact sensors may also improve detection and reporting accuracy.

## 5 Conclusion

In summary, this study underscores the importance and impact of standardized concussion protocols in professional soccer. The CFRP shows promise as a practical tool for improving athlete safety in South America, though its success hinges on continued investment in education, infrastructure, and regional adaptation. As awareness of concussion risks grows, ongoing research and cross-continental collaboration will be vital to ensuring player health remains a central priority in global sport.

## Data Availability

The original contributions presented in the study are publicly available. This data can be found here: https://data.mendeley.com/datasets/xdc29f7k44/1.

## References

[1] Demetriades AK Shah I Marklund N Clusmann H Peul W. Sport-related concussion in soccer—a scoping review of available guidelines and a call for action to FIFA & soccer governing bodies. Brain Spine. (2024) 4:102763. doi: 10.1016/j.bas.2024.102763, PMID: 38510627 PMC10951760

[2] Pierpoint LA Collins C. Epidemiology of sport-related concussion. Clin Sports Med. (2021) 40:1–18. doi: 10.1016/j.csm.2020.08.013, PMID: 33187601

[3] Levy ML Kasasbeh AS Baird LC Amene C Skeen J Marshall L. Concussions in soccer: a current understanding. World Neurosurg. (2012) 78:535–44. doi: 10.1016/j.wneu.2011.10.032, PMID: 22120567

[4] Kazl C Torres A. Definition, classification, and epidemiology of concussion. Semin Pediatr Neurol. (2019) 30:9–13. doi: 10.1016/j.spen.2019.03.003, PMID: 31235026

[5] CONMEBOL Medical Commission. Medical commission protocols. Luque: CONMEBOL (2024).

[6] Kimbler DE Murphy M Dhandapani KM. Concussion and the adolescent athlete. J Neurosci Nurs. (2011) 43:286–90. doi: 10.1097/JNN.0b013e31823858a6, PMID: 22045196 PMC3818791

[7] Clark M Guskiewics K. Translational research in traumatic brain injury. Boca Raton, FL: CRC Press (2016).

[8] Pangrazio O Forriol F Aguirre AS Beletanga MD Torres AR. Enhancing protocols for concussion management in professional soccer events. Cureus. (2024) 16:e64064. doi: 10.7759/cureus.64064, PMID: 39114186 PMC11304360

[9] Elbin RJ Sufrinko A Schatz P French J Henry L Burkhart S. Removal from play after concussion and recovery time. Pediatrics. (2016) 138:e20160910. doi: 10.1542/peds.2016-0910, PMID: 27573089 PMC5005026

[10] Asken BM McCrea MA Clugston JR Snyder AR Houck ZM Bauer RM. “Playing through it”: delayed reporting and removal from athletic activity after concussion predicts prolonged recovery. J Athl Train. (2016) 51:329–35. doi: 10.4085/1062-6050-51.5.02, PMID: 27111584 PMC4874376

[11] Echemendia RJ Meeuwisse W McCrory P Davis GA Putukian M Leddy J. The Sport Concussion Assessment Tool 5th edition (SCAT5): background and rationale. Br J Sports Med. (2017):848–50. doi: 10.1136/bjsports-2017-097506 28446453

[12] Pangrazio O Forriol F Pagura J Pedrinelli A Roby M Mazzoca G. Protocolo de Recomendaciones Medicas Competencias. Luque: CONMEBOL (2023).

[13] Abraham KJ Casey J Subotic A Tarzi C Zhu A Cusimano MD. Medical assessment of potential concussion in elite football: video analysis of the 2016 UEFA European championship. BMJ Open. (2019) 9:e024607. doi: 10.1136/bmjopen-2018-024607, PMID: 31147360 PMC6549745

[14] Cassoudesalle H Laborde B Orhant E Dehail P. Video analysis of concussion mechanisms and immediate management in French men’s professional football (soccer) from 2015 to 2019. Scand J Med Sci Sports. (2021) 31:465–72. doi: 10.1111/sms.13852, PMID: 33038045

[15] Ramkumar PN Navarro SM Haeberle HS Luu BC Jang A Frangiamore SJ. Concussion in American versus European professional soccer: a decade-long comparative analysis of incidence, return to play, performance, and longevity. Am J Sports Med. (2019) 47:2287–93. doi: 10.1177/0363546519859542, PMID: 31303010

[16] Vedung F Hänni S Tegner Y Johansson J Marklund N. Concussion incidence and recovery in Swedish elite soccer—prolonged recovery in female players. Scand J Med Sci Sports. (2020) 30:947–57. doi: 10.1111/sms.13644, PMID: 32100894

[17] Harmon KG Drezner JA Gammons M Guskiewicz KM Halstead M Herring SA. American Medical Society for Sports Medicine position statement: concussion in sport. Br J Sports Med. (2013) 47:15–26. doi: 10.1136/bjsports-2012-091941, PMID: 23243113

[18] Nilsson M Hägglund M Ekstrand J Waldén M. Head and neck injuries in professional soccer. Clin J Sport Med. (2013) 23:255–60. doi: 10.1097/JSM.0b013e31827ee6f8, PMID: 23348605

[19] Jo J Boltz AJ Williams KL Pasquina PF McAllister TW McCrea MA. Mechanisms of injury leading to concussions in collegiate soccer players: a CARE consortium study. Am J Sports Med. (2024) 52:1585–95. doi: 10.1177/03635465241240789, PMID: 38656160 PMC11823273

[20] Kristenson K Waldén M Ekstrand J Hägglund M. Lower injury rates for newcomers to professional soccer: a prospective cohort study over 9 consecutive seasons. Am J Sports Med. (2013) 41:1419–25. doi: 10.1177/0363546513485358, PMID: 23613443

